# Self- and peer assessment may not be an accurate measure of PBL tutorial process

**DOI:** 10.1186/1472-6920-8-55

**Published:** 2008-11-27

**Authors:** José Lúcio Martins Machado, Valéria Menezes Peixeiro Machado, Waldir Grec, Valdes Roberto Bollela, Joaquim Edson Vieira

**Affiliations:** 1 UNICID – Universidade Cidade de São Paulo Medical School, Rua Cesário Galeno 448/475, CEP 03071-000, São Paulo, Brazil

## Abstract

**Background:**

Universidade Cidade de São Paulo adopted a problem-based learning (PBL) strategy as the predominant method for teaching and learning medicine. Self-, peer- and tutor marks of the educational process are taken into account as part of the final grade, which also includes assessment of content. This study compared the different perspectives (and grades) of evaluators during tutorials with first year medical students, from 2004 to 2007 (n = 349), from seven semesters.

**Methods:**

The tutorial evaluation method was comprised of the students' self assessment (SA) (10%), tutor assessment (TA) (80%) and peer assessment (PA) (10%) to calculate a final educational process grade for each tutorial. We compared these three grades from each tutorial for seven semesters using ANOVA and a post hoc test.

**Results:**

A total of 349 students participated with 199 (57%) women and 150 (42%) men. The SA and PA scores were consistently greater than the TA scores. Moreover, the SA and PA groups did not show statistical difference in any semester evaluated, while both differed from tutor assessment in all semesters (Kruskal-Wallis, Dunn's test). The Spearman rank order showed significant (p < 0.0001) and positive correlation for the SA and PA groups (r = 0.806); this was not observed when we compared TA with PA (r = 0.456) or TA with SA (r = 0.376).

**Conclusion:**

Peer- and self-assessment marks might be reliable but not valid for PBL tutorial process, especially if these assessments are used for summative assessment, composing the final grade. This article suggests reconsideration of the use of summative assessment for self-evaluation in PBL tutorials.

## Background

The medical course of the Universidade Cidade de São Paulo (UNICID), in Sao Paulo, Brazil, adopted the problem-based learning (PBL) strategy as the predominant method for teaching and learning medicine since its opening in 2004. This choice was determined by the perspective that this pedagogy would improve students' critical thinking, communication skills, self-assessment skills and general professional competencies. Several changes have been introduced in medical education over the last 30 years, including the introduction of new contextualized approaches like PBL, the use of tools to enhance self-directed learning, the vertical integration of curriculum between basic and clinical sciences and the introduction of new formative and summative evaluation strategies that match with the curriculum changes [1].

Theoretically, PBL should encourage participants' self assessment as part of their learning and critical appraisal process. The use of self assessment by students and tutor rating of students' performances appear to be integral parts of many PBL tutorials similar self and tutor scores [1,2]. Moreover, a few reports have showed that students have a strong preference for peer feedback during the process of evaluation [3]. There has been little research, however, on self- and peer assessment in non-English speaking cultures.

Considering the potential interaction between grades and the learning environment with the application of peer feedback and self-assessment, this study aimed to compare self-, peer- and tutor assessments during PBL tutorials of first-year students in medicine as well as to research the reliability of self and peer assessments.

## Methods

The medical program extends for six years and it is organized for fifty students' groups divided in five tutorial cohorts, each of which meets twice a week during one semester. The curriculum stipulates three modules in each of the first eight semesters of the course, and more four additional semesters dedicated to internship. Each module normally contains eight problems developed in six weeks (total of 12 tutorial sessions). The students were trained to use the PBL (tutorials and assessment) during the first module (six weeks) denominated "Introduction to the Medicine" that focuses on history of medicine, ethics and bioethics'. The students are randomly organized in 6 groups and they stay together during one semester (3 modules). Every semester the students were re-arranged in new tutorial groups. The tutor for each group of students changed every module (six weeks).

The assessment in tutorials takes place at the end of every opening and closure of a problem. There are six pre-established criteria to be evaluated (skills to discuss and solve problems) by the tutor and the students, each of which can be rated from 1 to 5 (very bad to excellent), described below:

### Assessment form

Knowledge and ability to discuss the problem (minimum = 3, maximum = 15)

1.1 Identify problems and generate hypothesis

1.2 Make use of previous knowledge

1.3 Willing to participate with the group (member, coordinator, reporter)

Knowledge and ability to solve a problem (minimum = 3, maximum = 15)

1.4 Demonstrate previous studies by bringing pertinent information to the stated objective

1.5 Demonstrate ability to analyze and present organized information

1.6 Show analytical attitude related to the information presented and the group members' performance

The summative assessment in tutorials is added together as follows: tutor's assessment (80%), peer assessment (10%), and self assessment (10%) to compose the final score for each tutorial session. We evaluated seven classes (50 students/class) and the study sample included all first-year medical students who were registered at the UNICID from 2004 to the first semester of 2007. Students were asked to give verbal informed consent. Their identities were not disclosed during the study. This protocol was approved by CEP-UNICID (Research Ethical Board) registered at the National System for Ethics in Research (SISNEP) under number CAAE 0079.0.186.000-08. During the year 2004 and 2005 the analyzed marks from students and tutors were collected from freshmen students during the first five tutorials of module 1 and 2 and the first (1), middle (1) and final (1) tutorial of module 3 of each semester. From 2006 to 2007, five tutorials [first (2), middle (1) and final (2)] of each module were analyzed (Table 1). The following data were available for each student: self-mark, tutor-mark and the peer-mark. The median scores from tutorials during each semester for the self assessment (SA) and peer assessment (PA) were compared to the tutor awarded scores (TA). Trends in the scores were observed and interpreted in the context of tutorial assessment strategy. Data from SA, PA and TA were compared using ANOVA and a post hoc indicated test from a commercial statistical package.

**Table 1:** Number of tutorials analyzed in each of the 7 semesters – 2004 to 2007

Year	Module 1	Module 2	Module 3
2004 and 2005 (04 semesters)	Analyzed 05/12 tutorial sessions (first five)	Analyzed 05/12 tutorial sessions (first five)	Analyzed 03/10 tutorial sessions (first, middle and final)
2006 and 2007 1^st ^semester (03 semesters)	Analyzed 05/12 tutorial sessions (first, middle and final)	Analyzed 05/12 tutorial sessions (first, middle and final)	Analyzed 05/10 tutorial sessions (first, middle and final)

## Results

**Figure 1:**
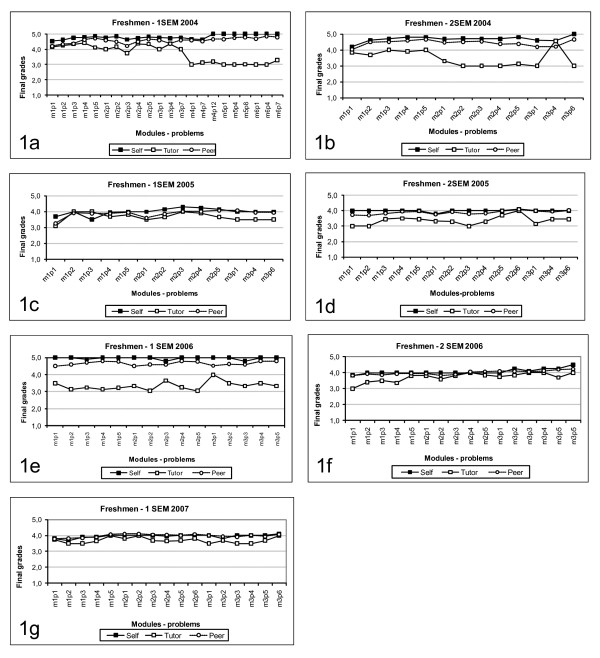
Self, Tutor and Peer evaluation in all semesters (medians).

A total number of 349 first year students participated with 199 (57%) women and 150 (42%) men. Students' self marks mean in each tutorial increased slightly every semester from the beginning to the end of the period, and was consistently observed in six of the seven semesters studied. (Figure 1a–g). The same trend was also observed with peers' marks. The data collected (TA, SA and PA) did not reach normal distribution using the Kolmogorov-Smirnov test. All data are presented as median and [25% – 75%] distribution. There was no statistically significant difference for the medians among self- and peer assessment.

In contrast to the SA and PA results, tutor marks showed a wide distribution that was quite different from the students' marks. In all seven semesters, the tutor marks were lower than the self- and peer-assessment marks. (Figure 1). The SA and PA groups did not show statistical difference for their medians in any of the seven semesters, while both were different from TA scores in all semesters (Kruskal-Wallis followed by Dunn's test), except the first semester in 2005 where PA did not differ from TA (figure 1c). The Spearman rank order showed significant positive correlation (p < 0.0001), higher for the SA and PA groups (r = 0.806) than for TA with either PA (r = 0.456) or SA (r = 0.376) (Table 2).

**Table 2:** Median score (25–75% range) and Spearman correlation.

	**Median score**	**25% – 75% range**	**Spearman r**
**Self-assessment – SA**	4.146	3.807 – 4.535	
**Tutor assessment – TA**	3.532	3.253 – 3.736	
**Peer assessment – PA**	4.059	3.728 – 4.415	
**SA × TA**			0.376
**PA × TA**			0.456
**SA × PA**			0.806

The medians within each group varied significantly from 2004 to 2007 (p < 0.001) (Kruskal-Wallis followed by Dunn's test). We can also see major differences in the TA scores from 1a and 1b and the rest of graphics (1c–1g). These differences seem to be just "noise" because our school was just beginning PBL in 2004 and was on a steep learning curve.

## Discussion

The medical programs that have implemented PBL have met with gains and difficulties as a result of the innovative traits of such a change. A number of challenges related to PBL implementation have to do with formative evaluation, which is an integral part of assessment in the horizontal and vertical modules of the program [4]. Assessment of the process and attitudes during tutorials sessions is supposed to embody PBL principles and is the central focus of student assessment [5]. Most of PBL schools report assessment during tutorials, but its purpose (summative or formative) is usually not obvious to students and faculty members. When stated, the use of assessment during tutorials is quite different, especially because it possesses psychometric shortcomings that limit their use in high-stake decision making [6].

We observed, from a cohort of seven semesters, that the grades that tutors awarded students were consistently lower than the grades the students awarded themselves and their peers. This may suggest a lack of transparency in evaluation procedures between students and tutors. We also could hypothesize that the scores from self- and peer-assessment seems to be reliable, but not necessarily valid, mainly because we could not observe a high correlation between tutor and self- or peer-assessment. This study has stimulated a reconsideration of the use of numerical scores for peer and self-assessment as components of the note that the students receive for their participation during the PBL tutorial sessions (summative).

The development of self-regulated learning is a major focus of problem-based learning programs. It has been shown that low-achieving students score themselves and their peers generously during medical school, although some high-achieving students may score themselves more harshly than faculty. According to that report, the PBL curriculum does not guarantee the appropriate development of self-assessment skills [7].

Self-assessment is an important formative component of PBL. This study demonstrated that the use of numerical self-assessment marks as part of the final grade for tutorial sessions contrasted sharply with scores provided by tutors despite of using the same criteria. Students score themselves generously, always above their tutor's marks. Their peer assessments followed suit, suggesting some sort of corporative effort toward increasing grades. The summative assessment could simplify the measurement of behavioral and cognitive skills related to content of tutorials, in addition to the supportive perception of students to the process of work group as a method of learning [4]. It is interesting to notice that summative assessment, when using self-awards, could not discriminate low or high achievers and the question remains whether it could discourage collaborative efforts or direct it only toward grades. It is also interesting to consider the dissimilarity from studies coming out of English culture, where students under-mark their own performance or equalize to the tutors', with the present results coming out of non English, more Latin culture [2,8-11]. These observations could be due to cultural differences (Latins being more polite, more community oriented *vs *individualistic as are US and Northern Europeans and Australians). Another possibility could be related to age/immaturity (Brazilian students go to medical school at the age of 17 to 18).

We analyzed about 1/3 overall tutorials, and during two years (2004/2005) we analyzed only the first five tutorials in module 1 and 2 compared with beginning, middle and final tutorials in other years. Considering that it takes time to develop "a group sense" or an intuitive perception of group pertaining, we might have some interference in final results. We also observe that scores seemed to come closer semester after semester and it could be explained by improvement of tutors' assessment skills with more PBL experience. On the other hand, students' self perception as a group can add a new factor in this equation, since their connection within and between their social networks may tend to imprint a value on their relationship (social capital). This value may play a role during the grade attribution, probably improving their own capacity for self and peer evaluation.

It is well known that assessment plays a large role in influencing student learning behavior. Therefore, it is important that the evaluation process do not hamper learning or adversely affect attainment of the goals of the curriculum. If student behaviors are directed toward achieving success on the evaluations, instructors' efforts to create a climate of self-directed learning and individual responsibility will be frustrated [12]. It would be reasonable to suggest that the use of numerical scores for self- and peer-assessment as part of the complete student grade allow a great risk of impairing the environment proposed by PBL tutorials [13]. There's no doubt that these methods address the major principles of PBL, however they possess psychometric shortcomings that limit their use in high-stake decision making [14].

The most addressed aspects or domains of teaching methodology for problem-based learning are content, cognitive processing, and group dynamics. There seems to be a low awareness of effective group dynamics during PBL tutorials as well as the absence of a mechanism for reflection that could assist groups analyze and learn from their behaviors [15,16]. As recommended by those authors, the UNICID applies regular and comprehensive training programs to instructors of PBL. This might have contributed to the stable and paralleled behavior observed from 2005 classes and on. However, the maintenance of marks originated from self-assessment to compose final grades might still underpin the tutorial environment. Whatever the evaluation a student may take from his tutor's mark to compose his/her final grade, there would be a trend for a generous self-assessment. This kind of self indulgence threatens the group productivity considering their articulation and planning for future sessions and more elaborated understanding, since these aspects could have been overwhelmed by the composition of final grades. The non-judgmental atmosphere of PBL tutorial groups could be compromised [17]. There are some limitations to this study that should be considered. The evaluation process during the sessions of PBL needs constant revision and training for all the newcomers, students or teachers. This process repeated a lot of times can create a climate of fatigue of that evaluation that can, for that matter, put in danger the attribution of grades. In addition, the strategy of problem-based learning is considered a new method among Brazilian higher education institutions and the students may need some time to acquire the ability to evaluate themselves in an impartial way.

We could also hypothesize that the lack of experience in PBL for students and tutors during the first one or two years of the program might have affected the TA, PA and SA results, and this was an inherent limitation in this new process. The follow up of future batches of students could make it clear for us, and we have been gathering more data to analyze it in future researches.

## Conclusion

Final grades considering self assessment marks may suggest to the tutorial participants a lack of transparency and impact as an inaccurate measure of performance. This study has stimulated reconsideration as to avoid the use of numerical scores for peer and self-assessment as part of the overall student grade during PBL tutorials.

• What is already known on this subject?

The use of self-assessment by students and tutor rating of students' performances are an integral part of assessing the educational process of PBL tutorials, and previous reports show similar self and tutor scores.

• What this study adds?

Summative self- and peer assessment might be reliable but not valid as part of the overall student grade during PBL tutorials, because we observed a clear difference among tutor and students' marks.

• Suggestions for further research

This study stimulated reconsideration of the use of peer- and self evaluation as part of the overall summative assessment of student performance during PBL tutorials.

## Competing interests

The authors declare that they have no competing interests.

## Authors' contributions

JLMM participated in the design of the study, evaluated the data and wrote the main text. VMPM evaluated the data and co-authored the main text. WG collected and evaluated the data. VRB evaluated the data and reviewed the text. JEV conceived of the study, performed the statistical analysis and reviewed the text. All authors read and approved the final manuscript

## Pre-publication history

The pre-publication history for this paper can be accessed here:


